# Clinical pregnancy outcomes with assisted reproduction in patients with 17α-Hydroxylase/17, 20-lyase deficiency: a single center cohort study and integrated analysis with reported cases

**DOI:** 10.3389/fendo.2026.1864787

**Published:** 2026-06-29

**Authors:** Ye Liu, Chunmei Zhang, Ran Lu, Rong Li, Ying Wang, Haining Wang

**Affiliations:** 1 Department of Endocrinology and Metabolism, State Key Laboratory of Female Fertility Promotion, Peking University Third Hospital, Beijing, China; 2 National Clinical Research Centre for Obstetrical and Gynecological Diseases, State Key Laboratory of Female Fertility Promotion, Ministry of Education Key Laboratory of Assisted Reproduction, Centre for Reproductive Medicine, Department of Obstetrics and Gynecology, Peking University Third Hospital, Beijing, China

**Keywords:** 17α-hydroxylase/17, 20-lyase deficiency, assisted reproductive technology, congenital adrenal hyperplasia, glucocorticoid therapy, pregnancy outcomes

## Abstract

**Background:**

17α-hydroxylase/17, 20-lyase deficiency (17OHD) represents a rare form of congenital adrenal hyperplasia. Affected 46, XX females typically present with sexual development abnormalities, endocrine disturbances and infertility. While assisted reproductive technology (ART) enables pregnancies in these patients, our knowledge about optimal management strategies and pregnancy outcomes remains limited.

**Methods:**

We conducted a retrospective cohort study of women with 17OHD who achieved clinical pregnancy at our center between January 2009 and August 2025, followed until December 2025. Moreover, we performed a systematic review of published cases, extracting and analyzing demographic, clinical, laboratory, treatment, and outcome data.

**Results:**

Among 20 female patients with 17OHD at our center, six desired fertility and three achieved clinical pregnancy with ART. Combined with 17 reported cases from 12 studies, a total of 20 pregnancies resulted in 23 live births. Median age at pregnancy preparation was 29 (range 21–42) years. The prevalence rates of hypertension and adrenal insufficiency were 25.0% (5/20) and 50.0% (10/20), respectively. Median follicular-phase progesterone was 20.8 nmol/L. Seventeen women conceived via frozen embryo transfer (FET) following ovarian stimulation, including nine with GnRH agonists (GnRHa), six with progestin-primed ovarian stimulation, one with GnRH antagonists, and one with GnRHa short protocol, respectively. Nearly 95% (18/19) participants received glucocorticoids before embryo transfer. Pregnancy complications (including gestational hypertension, diabetes, preeclampsia, and HELLP syndrome) occurred in 46.2% (6/13) cases. Only 25% (5/20) of the participants had term vaginal deliveries. All five offspring that were followed up developed normally.

**Conclusions:**

For women with 17OHD, a treatment protocol combining glucocorticoid-assisted progesterone suppression with FET represents a viable strategy to achieve pregnancy. However, such patients face high risks of adverse maternal and perinatal outcomes, requiring multidisciplinary management throughout gestation.

## 1 Introduction

Congenital adrenal hyperplasia (CAH) comprises a group of rare autosomal recessive disorders caused by adrenal steroidogenesis defects. Among these, 17α-hydroxylase/17, 20-lyase deficiency (17OHD) represents a rare subtype (1), induced by homozygous or compound heterozygous mutations in the *CYP17A1* gene. These mutations result in a complete or partial loss of 17α-hydroxylase/17, 20-lyase activity, leading to deficient synthesis of sex steroids and glucocorticoids, accompanied by increased mineralocorticoid levels. Affected 46, XX female patients typically present with abnormal sexual development, hypertension, hypokalemia, and infertility (1–3). With advances in diagnostic methods and assisted reproductive technology (ART), an increasing number of case reports have described women with 17OHD achieving live births through ART worldwide. However, optimal management strategies and potential pregnancy complications are not yet well-defined. Furthermore, evidence regarding the long-term health outcomes for both mothers and their children remains particularly scarce. In this study, we report the assisted reproductive courses and longitudinal follow-up of a small cohort of women with 17OHD treated at our center. By integrating previously reported cases of pregnancy, we aimed to delineate the reproductive challenges faced by women with 17OHD as well as to summarize and propose management strategies for this unique population.

## 2 Methods

### 2.1 Study design and participants

This retrospective cohort study was conducted at a tertiary referral center, including female patients diagnosed with 17OHD at our hospital between January 2009 and August 2025. The inclusion criteria were as follows: (1) confirmed diagnosis of 17OHD by clinical presentation and genetic testing, (2) chromosomal karyotype 46, XX, and (3) documented desire for fertility. Patients who achieved clinical pregnancy were followed up for at least one year postpartum. This study was approved by the Medical Science Research Ethics Commission of Peking University Third Hospital, and due to the retrospective design, informed consent was waived.

### 2.2 Clinical and laboratory assessments

Sex hormone levels were measured using a chemiluminescent immunoassay. Details of hormone assays are provided in the **Supplementary Material**. Genetic diagnosis was performed using second- or third-generation whole-exome sequencing. Adrenocorticotropic hormone (ACTH) stimulation tests were conducted to evaluate adrenal cortex function, with a peak cortisol level < 18 µg/dL indicating a blunted cortisol response (4). During the preconception period, patients received multidisciplinary management coordinated by endocrinologists and reproductive specialists at our center. Clinical pregnancy was defined as the presence of a gestational sac with a fetal heartbeat confirmed by pelvic ultrasound.

### 2.3 Reproductive management and outcomes

Based on the individual characteristics of patients, we implemented personalized ovarian stimulation protocols, followed by *in vitro* fertilization (IVF) and subsequent frozen-thawed embryo transfer (FET). The specific regimens include an ultra-long GnRH agonist (GnRHa) protocol, a GnRH antagonist (GnRHant) protocol, and an GnRHa short protocol for a patient with a previous failed FET cycle (See **Table 1** for the detailed protocols). Ovulation was triggered with 250 μg of recombinant human choriogonadotropin alpha (r-hCG, Ovidrel) when at least two dominant follicles reached 18 mm in diameter. Transvaginal ultrasound-guided oocyte retrieval was performed 36 hours later. The retrieved oocytes were fertilized via conventional IVF. All embryos and blastocysts were cryopreserved. For endometrial preparation prior to FET, a protocol combining glucocorticoids and GnRHa down-regulation was used to normalize serum progesterone levels, after which the cryopreserved embryos were thawed and transferred. Serum β-hCG levels were measured 14 days after embryo transfer. Clinical pregnancies received luteal phase support until 12 weeks of gestation. For non-local patients, remote follow-ups were conducted every three months during pregnancy, with in-person visits scheduled every 6 to 12 months postpartum.

**Table 1:** Assisted reproductive treatment protocols and outcomes of cases from our center.

Parameter	Case 1	Case 2	Case 3
Age​	34 years	24 years	30 years
Presenting History​	Infertility, elevated progesterone	Diagnosed at 19 years (hypertension, hypokalemia, oligomenorrhea); presented for fertility treatment	3-year history of infertility
*CYP17A1* genotype	c.1263G>A ^a^ c.1459_1467delGACTCTTTC ^a^	c.1169C>G ^a^ c.548G>A ^b^	c.1394T>C ^a, c^
Pretreatment for ART​	Dexamethasone 0.375 mg/day + long-acting GnRHa (2 months)	1. Dexamethasone 0.75 mg/day + long-acting GnRHa 2. Subsequently, Diane-35 was added	1. Dexamethasone 0.375 mg /day+ long-acting GnRHa 2. Subsequent cycle with Diane-35 pretreatment (short protocol)
COS Protocol​	Ultralong GnRHa protocol	GnRHant protocol	Cycle 1: ​ Ultralong GnRHa protocol Cycle 2: ​ Short protocol
Oocytes Retrieved​	10	18	Cycle 1:​ 10 Cycle 2:​ 12
Embryology Outcome​	6 embryos (8G1 ×1, 8G2 ×4, 10G2 ×1)	10 embryos (8G1 ×5, 8G1- ×1, 8G2 ×2, 7G1- ×2) Blastocysts:​ 2 (D5 4AB* ×1, D6 4BC ×1)	Cycle 1:​ 1 embryo (8G2) Cycle 2:​ 2 embryos (8G2 ×2)
Embryo Transfer​	8G1 ×1, 5G1 ×1	8G1 ×2	8G2 ×2
Use of Glucocorticoids During Pregnancy	Dexamethasone was discontinued at 7 weeks of gestation.	Prednisone	Prednisone was discontinued at 7 weeks of gestation.
Pregnancy Outcome​	Singleton live birth at 37 weeks (healthy male, 2.94 kg)	Singleton live birth at 34 weeks via emergency cesarean section (male, 2.15 kg)	Singleton pregnancy with embryonic demise at 16 weeks (normal embryo CNV)
Pregnancy Complications​	HDP, GDM	HDP (preeclampsia), GDM	HDP, Refractory hypokalemia in early pregnancy
Postpartum/Follow-up​	Medication stopped; normal blood pressure and electrolytes; regular menses; normal child development at 6 years	Continued prednisone 5 mg/day+ amlodipine; ovarian cysts resolved with OCs and having regular menses; normal child development at 4 years	Blood pressure and potassium normalized after termination; regular menses resumed

^a^Pathogenetic; ^b^ Likely pathogenetic; ^c^, Whole-exome next-generation sequencing and third-generation sequencing both identified this heterozygous pathogenic mutation in the *CYP17A1* gene. Although a second mutation has not been identified and her parents were not willing to get tested, a diagnosis of 17OHD was made based on the characteristic clinical manifestations of this case. ART, assisted reproductive technology; COH, controlled ovarian hyperstimulation; CNV, copy number variants; GDM, gestational diabetes mellitus; GnRHa, gonadotropin-releasing hormone agonist; GnRHant, gonadotropin-releasing hormone antagonist; HDP, hypertensive disorders of pregnancy OCs, Oral Contraceptives.

### 2.4 Literature review and data synthesis

To place our findings in context within the existing body of evidence, we performed a systematic literature search on PubMed, the Web of Science, Embase as well as in the China National Knowledge Infrastructure and Chinese Medical Ace Base from database inception to May 20, 2025. The search strategy and inclusion diagram of previous studies are detailed in the **Supplementary Material**. The literature search and review followed PRISMA 2020 guidelines (5) and studies reporting pregnancy outcomes in women with 17OHD were identified.

### 2.5 Data collection and statistical analysis

Demographic data, clinical manifestations, laboratory findings, treatment protocols, and pregnancy outcomes were extracted both for our institutional cohort and previously reported cases using a standardized data collection form. Data were independently verified by two physicians, with adjudication by a third researcher in cases of disagreement. Descriptive statistics were used to summarize clinical characteristics. Continuous variables were expressed as medians with ranges (minimum−maximum), and categorical variables as frequencies (percentages). All data were collected until December 31, 2025.

## 3 Results

### 3.1 Patient disposition and baseline characteristics

**Figure 1:**
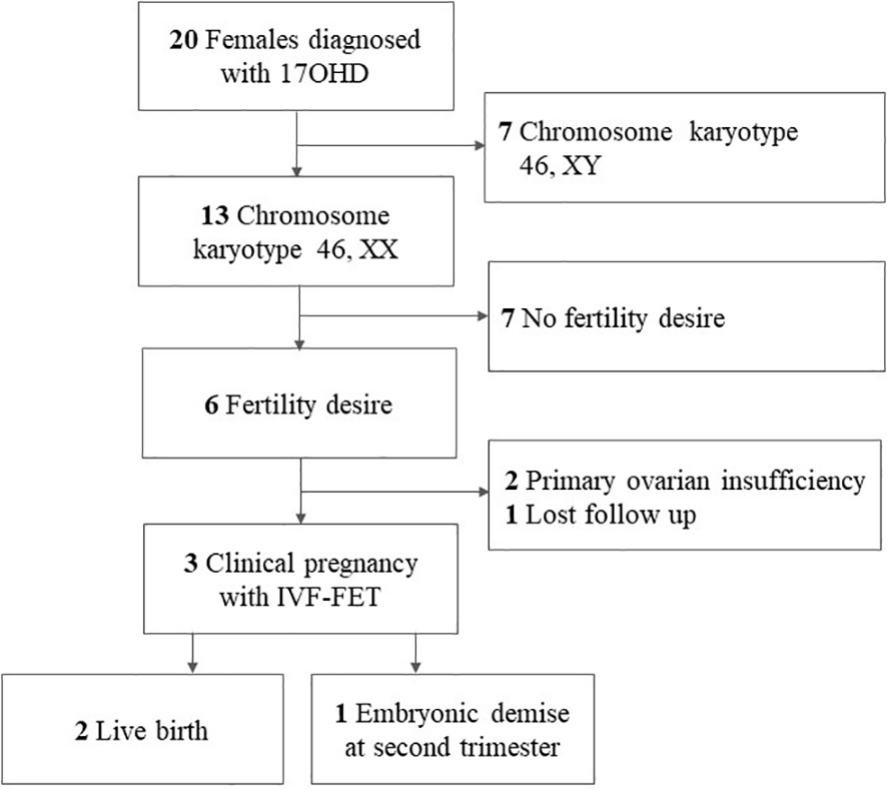
Inclusion flowchart of cases in our center. 170HD, 17α-Hydroxylase/17, 20-lyase Deficiency; IVF-FET, in vitro fertilization frozen embryo transfer.

Between January 2009 and August 2025, 20 female patients were diagnosed with 17OHD at our hospital. Among them, six expressed a desire for fertility, three of whom achieved clinical pregnancy. Of the remaining three, two patients were diagnosed with premature ovarian insufficiency and one was lost during the follow-up. All three pregnant patients completed follow-up through December 2025 (**Figure 1**). Our systematic literature review identified 12 eligible studies (**Supplementary Figure 1**) (6–17), reporting an additional 17 women with 17OHD who successfully delivered live infants. **Supplementary Tables 1** and **2** summarizes the clinical characteristics, ART courses, pregnancy outcomes, and follow-up data for all 20 women (3 from our center and 17 from the literature).

### 3.2 Reproductive management and outcomes of the institutional cohort

The three patients from our center who achieved clinical pregnancy were aged 24–34 years at the time of treatment initiation. Their clinical manifestations, diagnostic processes and genotypes were presented with details in the **Supplementary Material**. The total number of oocytes retrieved per cycle ranged from 10 to 18, and the number of cryopreserved embryos per patient ranged from 1 to 10. All three patients have cryopreserved embryos available for subsequent thawing and transfer. Detailed individualized ART protocols for these three patients are presented in **Table 1**.

Prior to FET, glucocorticoids were administered to all three patients—either alone or in combination with a GnRHa—to control progesterone levels, which were successfully maintained between 0.77 and 2.47 nmol/L. Following this, two patients achieved clinical pregnancy after the first FET cycle. The third patient had an initial failed cycle but subsequently achieved a successful pregnancy in a later transfer.

Pregnancy outcomes varied in the three patients. One patient had an uncomplicated full-term delivery at 37 weeks via cesarean section, delivering a healthy male infant. A second patient experienced a pregnancy complicated by gestational diabetes and preeclampsia requiring emergency cesarean delivery at 34 weeks. Her infant son developed neonatal pneumonia after birth, was managed in the neonatal intensive care unit for one week, and was discharged safely. The third patient developed refractory hypokalemia and untreated hypertension during early pregnancy, resulting in intrauterine fetal demise at 16 weeks of gestation. The metabolic abnormalities of the third patient resolved following pregnancy termination.

Postpartum follow-up revealed varied gynecologic outcomes. Two patients resumed regular menstrual cycles after delivery, while one patient developed bilateral ovarian cysts requiring management with oral contraceptive. Long-term offspring follow-up for the two live births demonstrated healthy growth and development at ages 6 and 4 years, respectively.

### 3.3 Integrated analysis of treatment protocols and reproductive outcomes among all reported patients

#### 3.3.1 Characteristics of women with 17OHD who achieved pregnancy

**Supplementary Table 1** presents the characteristics of women with 17OHD who have been documented to achieve pregnancy. Together with our three cases, a total of 20 women with 17OHD have been described with achieved clinical pregnancies, resulting in 23 live births. The median age at pregnancy preparation was 29 (21–42) years (n=19). Among these women, 13/20 (65.0%) had a history of spontaneous menstruation, with a median age at menarche of 14 (12–18) years (n=12). Hypertension was present in 5/20 (25.0%) of cases and adrenal insufficiency or impaired adrenal reserve was documented in 10/20 (50.0%) of the patients. Related to ovarian reserve markers, the median levels were as follows: follicle-stimulating hormone (FSH) 7.1 (3.3–26.8) mIU/mL (n=18), luteinizing hormone (LH) 7.8 (2.8–28.5) mIU/mL (n=19), progesterone 20.8 (6.7–100.2) nmol/L (n=18), and anti Müllerian hormone (AMH) 2.77 (1.43–13.35) ng/mL (n=10). Among those with reported pelvic imaging, 6/11 (54.5%) and 12/16 (75%) displayed a small uterus and multiple ovarian cysts, respectively.

#### 3.3.2 Assisted reproductive treatment protocols

**Figure 2:**
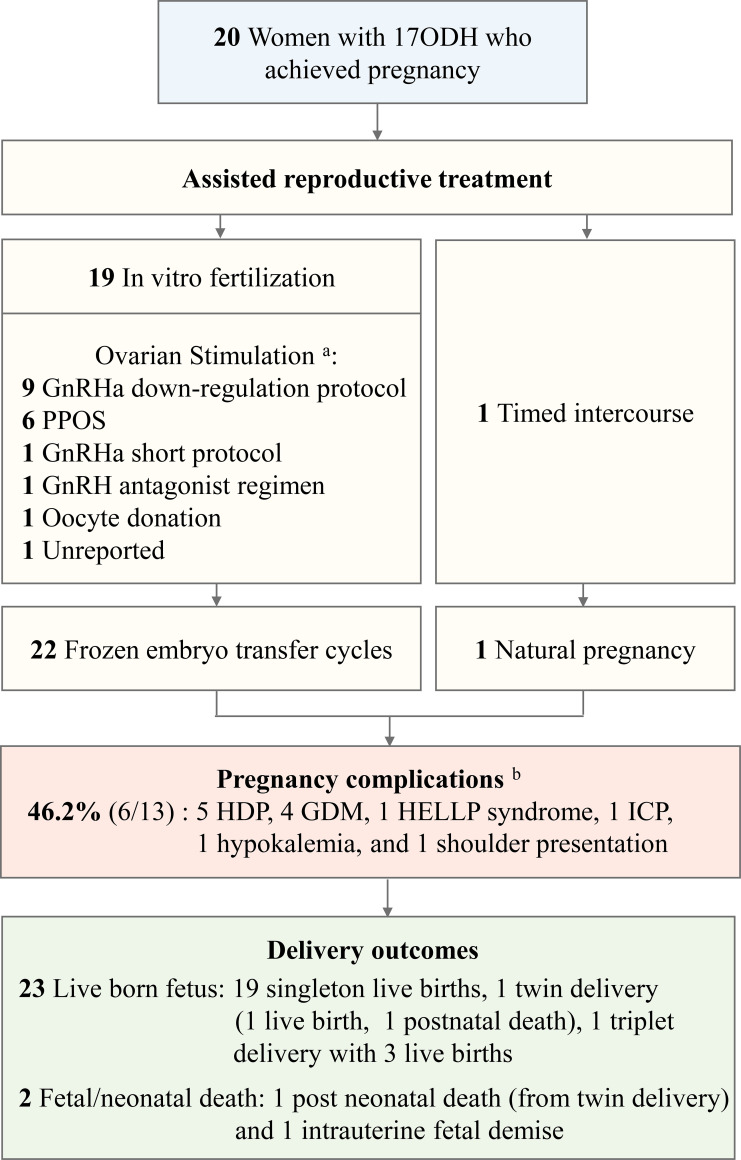
Assisted reproductive treatment and fertility outcomes in women with 170HD **(a)** The ovarian stimulation protocol for cycles achieving clinical pregnancy; **(b)** Analysis in cases with detailed reporting of the pregnancy course, including those with multiple complications. 170DH, 17α-Hydroxylase/17, 20-lyase Deficiency; GDM, gestational diabetes mellitus; GnRHa, gonadotropin-releasing hormone agonist; HELLP, hemolysis, elevated liver enzymes, low platelets; HDP, hypertensive disorders of pregnancy; ICP, Intrahepatic Cholestasis of Pregnancy; PPOS, Progestin-primed ovarian stimulation.

Concerning the ART protocols (**Figure 2**; **Supplementary Table 2**), 19 patients achieved pregnancy by IVF, and one patient achieved natural pregnancy following ovulation monitoring and timed intercourse. Regarding oocyte origin for IVF, one pregnancy was achieved using donated oocytes, and in one case the conception method was not specified. Pregnancy was achieved via ovarian stimulation-IVF-FET in 17 of the 20 women (85.0%). The ovarian stimulation protocols included: long-acting GnRHa (9/17, 52.9%), progestin-primed ovarian stimulation (PPOS) (6/17, 35.3%), GnRH antagonist (n=1), and short protocol (n=1). Prior to embryo transfer, 18/19 (94.7%) women received glucocorticoids, and 10/19 (52.6%) also underwent GnRHa downregulation. Progesterone levels prior to embryo transfer were controlled within the range of < 0.64–3.34 nmol/L.

#### 3.3.3 Pregnancy and neonatal outcomes

Among the 13 women with detailed pregnancy course data, 6/13 (46.2%) experienced pregnancy complications, including HELLP (hemolysis, elevated liver enzymes, low platelets) syndrome, embryonic demise, preeclampsia, gestational diabetes mellitus (GDM), and intrahepatic cholestasis of pregnancy. Of the 20 deliveries, only 5/20 (25%) were term spontaneous vaginal deliveries; the remaining 15 were complicated by preterm birth, cesarean section, cervical balloon induction, or postpartum hemorrhage. Among the 23 live-born offspring, five were followed for at least one year, all demonstrating healthy growth and development.

## 4 Discussion

17OHD is a rare subtype of CAH characterized by the dysfunction of 17α-hydroxylase/17, 20-lyase. Affected 46, XX females typically present with a constellation of hypertension and hypokalemia resulting from excess mineralocorticoid production, alongside primary amenorrhea or oligomenorrhea due to impaired estrogen synthesis and anovulation (1). The characteristic hormonal profile includes elevated progesterone, low estradiol, and variable gonadotropin levels, often accompanied by the development of multiple ovarian cysts driven by persistently elevated FSH. Despite apparently normal external genitalia at birth, affected individuals exhibit absent or delayed pubertal development, with many remaining undiagnosed until infertility investigation in adulthood. The clinical phenotype varies with the degree of enzymatic impairment. Historically, fertility has been considered extremely unlikely in women with this condition. However, due to advances in diagnostic methods and ART, a total of 20 pregnancies have been reported in women with 17OHD, resulted in 23 live-born and infants. By synthesizing these cases, we analyzed data on assisted reproduction, pregnancy management, and long-term follow-up in women with 17OHD, providing a comprehensive and in-depth understanding of the effects of this condition on female reproductive health.

### 4.1 Assisted reproductive strategies for women with 17OHD

#### 4.1.1 Genetic consulting

Genetic counseling should be considered for patients with 17OHD before pregnancy, since all offspring of affected women are obligate carriers of a *CYP17A1* mutation. Although the pathogenetic mutation rate of *CYP17A1* in the general population is much lower than that of other CAH genes, such as *CYP21A2* (1), genetic screening of the spouse and genetic counseling for the couple have clinical significance for the long-term health follow-up of their offspring. If, unfortunately, the husband also carries a pathogenic mutation in *CYP17A1*, multidisciplinary counseling including a geneticist and preimplantation genetic testing may represent a strategy to reduce the risk of severe genetic disorders in offspring.

The use of prenatal diagnosis and dexamethasone treatment in other forms of CAH, such as 21-hydroxylase deficiency, remains controversial, although they are sometimes employed to prevent virilisation of the external genitalia in affected female fetuses (18). However, testosterone synthesis is impaired in 17OHD. Therefore, neither prenatal diagnosis nor prenatal treatment is necessary. However, if both spouses carry a pathogenic mutation in *CYP17A1* during preconception genetic counseling, the neonate’s genotype and clinical phenotype should be monitored after birth, with long-term follow-up.

#### 4.1.2 Assisted reproductive protocol

Successful pregnancy could be conceptualized as a three-stage process: endometrial preparation, oocyte/embryo quality, and embryo implantation. Historically, women with 17OHD face infertility due to multiple factors: chronically low estrogen levels resulting in uterine hypoplasia, disrupted folliculogenesis causing anovulation, and persistently elevated progesterone levels, which may compromise endometrial receptivity and hinder embryo implantation (1, 3, 4). With improved awareness of 17OHD, advances in diagnostic techniques, and greater access to testing, patients with 17OHD are now diagnosed earlier and more frequently than previously. The presence of a uterus and normal AMH levels in most participants indicates preserved ovarian reserve and reproductive potential. This preserved potential suggests that targeted treatment addressing these three key stages could enable women with 17OHD to achieve pregnancy.

At baseline, 54.5% of participants presented with a reduced uterine size. This was treated with several months of artificial cycle therapy to promote uterine development, which was followed by adequate estrogen and progesterone support around embryo transfer to help establish a receptive endometrium.

Second, effectively obtaining viable oocytes remains a key challenge. Encouragingly, in the reviewed literature, 85% of women with 17OHD were able to produce mature oocytes and generate high-quality embryos through ovarian stimulation, underscoring their reproductive potential. A major obstacle is the presence of persistently elevated gonadotropin and progesterone levels prior to stimulation, unless suppressed, may lead to poor follicular development and cycle cancellation. To address this, glucocorticoids combined with long-acting GnRHa (sometimes with oral contraceptives) can be used to suppress adrenal and ovarian function, respectively, thereby lowering pre-stimulation progesterone levels. In our study, most patients successfully obtained embryos using this approach alongside controlled ovarian hyperstimulation (COH), indicating favorable outcomes. An alternative is the PPOS protocol, employed in six women here, which uses progestin to prevent premature LH surges. PPOS offers a more flexible and economical regimen as it requires no down-regulation and can be initiated at any time. Several studies report comparable oocyte yields between PPOS and conventional (antagonist or agonist) protocols (19–22). However, due to the limited number of 17OHD cases, the safety and efficacy of PPOS relative to GnRHa protocols require further validation.

Persistently elevated progesterone also disrupts the third stage—embryo implantation—by causing endometrial-embryo asynchrony. Under physiological conditions, finely-tuned cyclic changes in progesterone levels are crucial for ovulation, fertilization, embryo implantation, and maintenance of pregnancy (23). However, in women with 17OHD, impaired glucocorticoid and estrogen synthesis leads to increased pituitary secretions of ACTH and gonadotropins via feedback mechanisms. This, in turn, stimulates the adrenal glands and ovaries such that progesterone is continuously synthesized and secreted. As a result, persistently increased non-cyclic progesterone levels establish a “contraceptive barrier.” Furthermore, in ART cycles, progesterone level increase on the day of hCG administration could lead to asynchrony between the endometrium and embryo, reduced endometrial receptivity, and premature closure of the implantation window. Consequently, pregnancy rates in fresh cycles are markedly reduced (24). Therefore, apart from one case of natural pregnancy following ovulation monitoring, all pregnancies in the reported cases of women with 17OHD were achieved after FET. In this study, prior to FET, low-dose glucocorticoids were continued in 94.7% of patients to suppress adrenal-derived progesterone. Concurrently, ovarian-derived progesterone was suppressed using oral contraceptives, occasionally combined with a long-acting GnRHa. This dual suppression strategy achieves optimal endometrial-embryo synchronization. Adequate estrogen and progesterone supplementation after embryo transfer further ensures successful embryo implantation. Therefore, the strategy of ovarian stimulation followed by embryo cryopreservation and FET under dual adrenal and ovarian progesterone suppression represents a feasible pathway to assisted reproduction in women with 17OHD.

### 4.2 Challenges and management in pregnant women with 17OHD

Although ART offers conception opportunities for patients with 17OHD, the pathophysiological changes during pregnancy can exacerbate the existing metabolic disorders, making pregnancy management another major challenge. First, once pregnancy is confirmed, continued suppression of elevated progesterone levels is no longer necessary. Therefore, dexamethasone should be either discontinued promptly after confirming pregnancy or switched to prednisone or hydrocortisone to prevent potential adverse effects on the fetus (18, 25). Second, the inherent mineralocorticoid excess in 17OHD establishes a pathophysiological basis for hypertension and electrolyte disturbances. For example, in our study, the patients in cases 1 and 3 developed new-onset hypertension during pregnancy despite previously normal blood pressure, while the patient in case 2 experienced a hypertensive crisis during late pregnancy even with regular antihypertensive medication and close monitoring. In addition, the patient in case 3 developed severe refractory hypokalemia during pregnancy, and the hypokalemia resolved spontaneously after delivery. These results suggest that pregnancy-related changes in steroid hormone levels exacerbate or trigger blood pressure and electrolyte abnormalities in women with 17OHD. Therefore, even in patients with normal blood pressure and electrolyte levels prior to pregnancy, careful monitoring and management of blood pressure and electrolytes throughout pregnancy are essential. Another pathological feature of 17OHD is impaired adrenal glucocorticoid synthesis or impaired adrenal reserve. Among all the reported cases of pregnant women with 17OHD summarized above, 76.9% involved known adrenal insufficiency or impaired adrenal reserve. For women with known clinical adrenal insufficiency, glucocorticoid replacement should be maintained throughout pregnancy. This approach also helps to control hypertension resulting from mineralocorticoid excess. The glucocorticoid dosage should be increased as appropriate during late pregnancy (1). For those with only impaired adrenal reserve, stress-dose glucocorticoids (such as a temporary intravenous infusion of hydrocortisone at stress doses) should be administered during labor to prevent adrenal crisis, and the pre-pregnancy regimen could be resumed after delivery. A considerable proportion of live births were complicated by adverse maternal and perinatal outcomes (e.g., hypertensive disorders, HELLP syndrome, stillbirth, fetal distress), and only 25% culminated in a full-term vaginal delivery. This indicates a substantially elevated risk profile in 17OHD pregnancies. Given the current lack of consensus guidelines, we recommend implementing a structured, multidisciplinary management plan involving endocrinology and obstetrics teams to optimize care and improve maternal and neonatal outcomes.

### 4.3 Long-term management of reproductive health in women with 17OHD

Even if the delivery is successful, the long-term reproductive health management of the patient still faces challenges. Menstrual abnormalities are a persistent issue for women with 17OHD and might manifest as amenorrhea, oligomenorrhea, or prolonged spotting. These abnormalities are associated with impaired estrogen synthesis, persistently elevated progesterone levels, dysregulated gonadotropin secretion, and irregular ovulation. Artificial cycle therapy could supplement estrogen and suppress persistently elevated gonadotropin and progesterone levels, which should theoretically help regulate menstrual cycles in these women. However, in clinical practice, 45.4% of women with 17OHD in our center failed to maintain regular menstrual cycles despite receiving commonly used artificial cycle regimens such as estradiol plus cyclic dydrogesterone, Femoston 2/10, or Diane-35 (data not shown). Future studies are needed to explore and summarize effective menstrual management strategies for women with 17OHD.

Ovarian cysts are also a common complication in women with 17OHD, with an incidence as high as 75%. The development of ovarian cysts is associated with persistently elevated FSH levels. These cysts are typically persistent or recurrent, and could reach a considerable size with the risk of rupture or torsion. Management primarily involves artificial cycle therapy to reduce their size. Given that surgery may diminish ovarian reserve, a thorough endocrine evaluation—rather than immediate surgical intervention—should be the first step upon detection of such cysts. Surgical management is thus indicated solely for suspected malignancy or emergent complications (e.g., rupture or torsion). However, in some patients who are not diagnosed with 17OHD in a timely manner, ovarian cysts might be misdiagnosed and treated with surgical intervention. For example, the sister of the patient in Case 2, who shared the identical phenotype and genotype, initially underwent cystectomy at another hospital for bilateral ovarian cysts. Due to the lack of postoperative artificial cycle therapy, her cysts recurred. After being diagnosed at our hospital and receiving artificial cycle therapy, her cysts decreased in size. This case underscores the need to improve gynecologists’ recognition of 17OHD in order to prevent unnecessary surgical interventions.

Among women with 17OHD who experienced fertility outcomes, AMH levels were within the optimal range in all cases for which data were available. However, in our center, two female patients were diagnosed with premature ovarian insufficiency at the ages of 30 and 31 years, respectively, both with AMH levels both below 0.1 ng/mL. Similarly, a previous cohort study of patients with 21-hydroxylase deficiency reported that 4 of the 11 young women with 21-hydroxylase deficiency had markedly decreased AMH levels (26). Although there is currently no definitive evidence that CAH could accelerate ovarian insufficiency, women with CAH already have reduced fertility potential. As an increasing number of patients with 17OHD or other forms of CAH are being diagnosed during adolescence, we recommend that AMH levels be assessed at the initial visit. For those interested in future fertility, ovarian reserve evaluation and counseling are also advised. These early interventions are key to supporting informed reproductive decisions.

### 4.4 Strengths and limitations

In this study, we provide a detailed description of the assisted reproductive processes and postpartum follow-up in three women with the rare condition, 17OHD. Moreover, we comprehensively reviewed all reported cases of women with 17OHD with available fertility outcomes, and summarized a feasible assisted reproductive protocol. This involves ovarian stimulation (using either a PPOS or a conventional [non-PPOS] protocol) followed by FET. Furthermore, we systematically analyzed the reproductive health challenges encountered by women with 17OHD across the entire life course. The study highlights key aspects in pregnancy management for these women and introduces the concept of fertility preservation. However, this study retains certain limitations. First, pregnancy in patients with 17OHD are exceptionally rare. Consequently, our single-center cohort comprises only three cases, and most available reports in the literature are individual case descriptions. Given the limited sample size, robust statistical comparisons and meta-analysis were not feasible. Our findings should thus be considered preliminary rather than conclusive. Second, among previously reported cases, half lacked data on AMH levels, 35% did not document pregnancy management details, 80% did not report on long-term follow-up data for offspring, and only five described biochemical markers related to mineralocorticoid excess. Furthermore, preconception management strategies varied across studies. It is currently uncertain which glucocorticoid regimen most effectively controls elevated progesterone, or whether GnRHa-based COH or PPOS is preferable for ovarian stimulation, owing to insufficient data to resolve these questions. Consequently, our understanding of reproductive potential, optimal ART strategies, pregnancy risks, and long-term maternal and offspring health in 17OHD remains limited. Therefore, multicenter prospective studies are needed to accumulate robust data, better define the reproductive health challenges in this population, and establish evidence-based management strategies.

## 5 Conclusion

17OHD is a rare genetic disorder that severely compromises female reproductive health. Data from our single center cohort and other successful cases indicate that ART based on glucocorticoid therapy represents a feasible pathway to parenthood. Nevertheless, pregnancies in these patients are associated with significant risks of developing maternal and fetal complications, such as hypertensive disorders, GDM, and preterm birth. Beyond fertility, long-term management challenges such as menstrual disorders and ovarian cysts require dedicated attention. Further studies are needed to address unmet needs in menstrual regulation and fertility assessment. Therefore, establishing a robust multidisciplinary framework involving endocrinology, reproductive medicine, and obstetrics is crucial to optimizing lifelong reproductive health for women with 17OHD.

## Data Availability

The original contributions presented in the study are included in the article/**Supplementary Material**. Further inquiries can be directed to the corresponding authors.
