# Supplementary material for: Clinical pregnancy outcomes with assisted reproduction in patients with 17α-Hydroxylase/17, 20-lyase deficiency: a single center cohort study and integrated analysis with reported cases
Source: Front Endocrinol (Lausanne). 2026 Jun 29;17:1864787. doi: 10.3389/fendo.2026.1864787 (PMC13357200; doi:10.3389/fendo.2026.1864787)
Supplement: Supplementary file 1 [file DataSheet1.docx]

**Supplementary materials**

1. **Sex hormone assays**
2. **Search strategies for literature review**
3. **Figure S1. Flow diagram of reported cases inclusion.**
4. **Table S1.** **Characteristics of 20 cases with 17α-Hydroxylase/17,20-lyase at administration**.
5. **Table S2. Assisted reproduction, reproductive and offspring outcomes in 20 patients with 17α-Hydroxylase/17, 20-lyase**.
6. **Clinical manifestations and diagnostic processes of the three patients from our center**
7. **Table S3. Genotypes of the three patients from our center**

# 1. Sex hormone assays

Serum FSH, LH, and estradiol levels were measured by chemiluminescent immunoassays (Beckman Coulter, Suzhou, China). Serum progesterone and AMH were measured by chemiluminescent immunoassays (Kangrun Biotech, Guangzhou, China). The assay range and inter-assay coefficient of variation were as follows: 0.2−200 mIU/mL and 5.4% for FSH, 0.2−250 mIU/mL and 4.3% for LH, 15−5200 pg/mL and 10% for estradiol, 0.32−190.8 ng/mL and 8.0% for progesterone, and 0.01−30 ng/mL and 8.0% for AMH, respectively.

# 2. Search strategies for literature review

PubMed, the Web of Science, Embase, China National Knowledge Infrastructure and Chinese Medical Ace Base were searched from inception to May 2025 using the following search strategy:

**Pubmed**

1. Search (17α-hydroxylase or 17α-Hydroxylase/17,20-lyase or 17OH or 17α-hydroxylase/ 17,20-lyase deficiency) for [Title/Abstract].

2. Search (pregnant or pregnancy or birth) for [Title/Abstract].

3. Search #1 and #2.

**Web of Science**

1. Search: pregnant (Topic) or pregnancy (Topic) or birth (Topic)

2. Search: 17α-hydroxylase (Topic) or 17α-Hydroxylase/17,20-lyase (Topic) or 17OH (Topic) and 17α-hydroxylase/ 17,20-lyase deficiency (Topic)

3. Search #1 and #2

**Embase**

1. Search: '17α-hydroxylase' OR '17α-hydroxylase/17,20-lyase' OR '17oh' OR '17α-hydroxylase/ 17,20-lyase deficiency':ab,ti

2. Search: 'pregnant' OR 'pregnancy'/exp OR 'pregnancy' OR 'birth':ab,ti

3. Search: #1 and #2

**China National Knowledge Infrastructure 中国知网**

1. Search:（篇关摘：17α-羟化酶(模糊)）OR（篇关摘：17α-羟化酶/17,20-裂解酶(模糊)）OR（篇关摘：17OHD(模糊)）OR（篇关摘：17α-羟化酶/17,20-裂解酶缺乏症(模糊)）

2. Search:（篇关摘：妊娠(模糊)）OR（篇关摘：分娩(模糊)）OR（篇关摘：怀孕(模糊)）OR（篇关摘：出生(模糊)）

3. Search: #1 and #2

**Chinese Medical Ace Base 中国生物医学文献服务系统**

1. Search: "17α-羟化酶"[摘要:智能] OR "17α-羟化酶/17,20-裂解酶"[摘要:智能] OR "17OHD"[摘要:智能] OR "17α-羟化酶/17,20-裂解酶缺乏症"[摘要:智能]

2. Search: "妊娠"[摘要:智能] OR "分娩"[摘要:智能] OR "怀孕"[摘要:智能] OR "出生"[摘要:智能]

3. Search: #1 and #2

# 3. Figure S1. Flow diagram of reported cases inclusion.


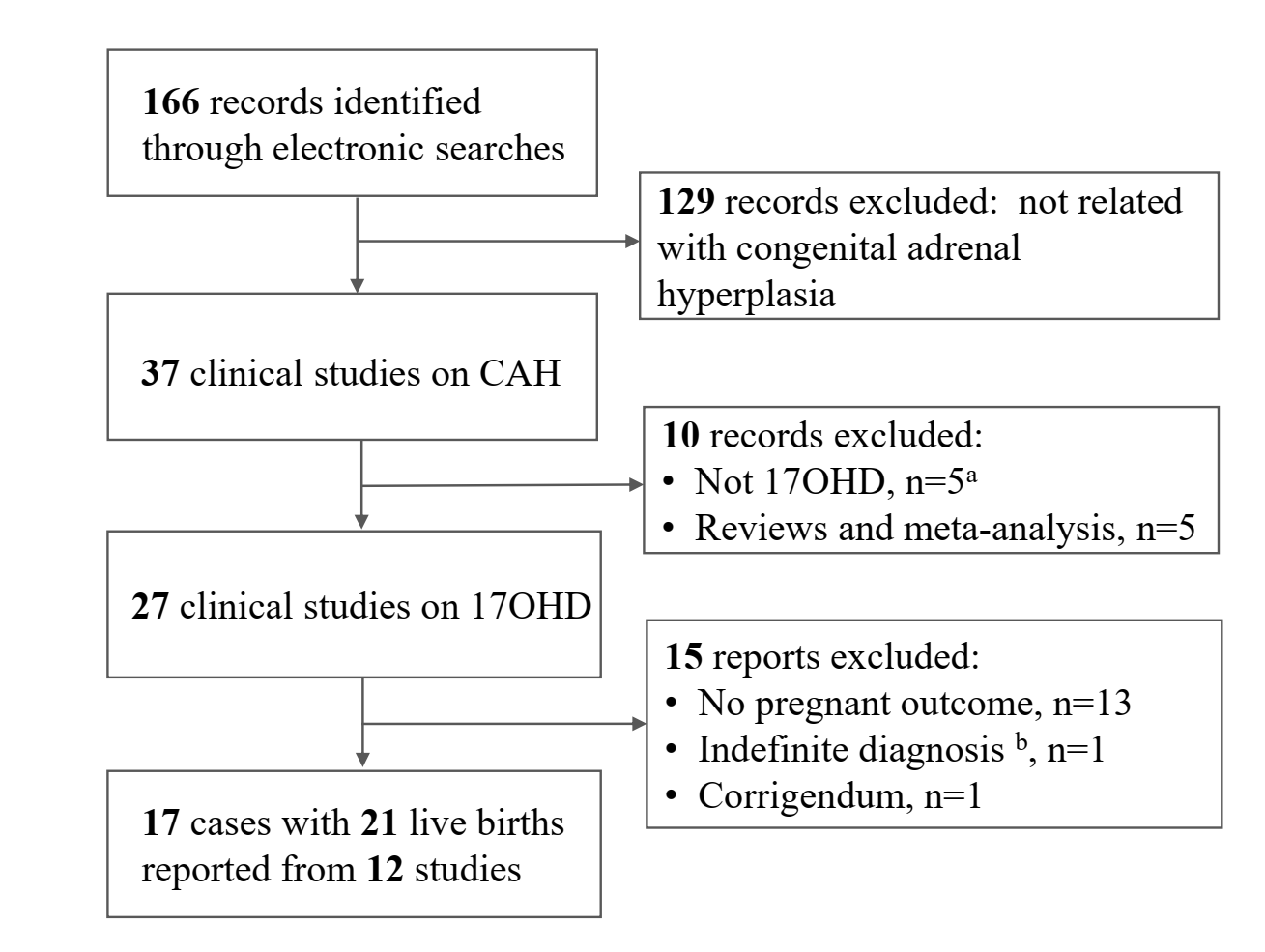


CAH, congenital adrenal hyperplasia; 17OHD, 17α-hydroxylase/17,20-lyase deficiency

a. Including 21-hydroxylase deficiency, cytochrome P450 oxidoreductase deficiency, and isolated 17,20-lyase deficiency; b. Gene analysis of the patient did not identify any mutation related with congenital adrenal hyperplasia

# 4. Table S1 Characteristics of 20 cases with 17α-hydroxylase/17,20-lyase at administration.

| **Case** | **Author and Publication year** | **Age ^a^**  **year-old** | **Spontaneous menarche**  **year-old** | **Hypertension** | **Adrenal insufficiency ^b^** | **FSH mIU/mL** | **LH mIU/ml** | **Progesterone**  **nmol/L** | **AMH ng/mL** | **Uterus** | **Ovaries** |
| --- | --- | --- | --- | --- | --- | --- | --- | --- | --- | --- | --- |
| 1 | Our center | 34 | 18 | No | Yes | 6.6 | 9.2 | 10.9 | 2.20 | Normal | Cysts |
| 2 | Our center | 24 | 12 | Yes | Yes | 3.8 | 7.1 | 52.0 ^c^ | 1.79 | Small | Cysts |
| 3 | Our center | 30 | 14 | No | No | 8.1 | 14.3 | 20.8 | 3.85 | Normal | Cysts |
| 4 | Ben-Nun I ^(6)^  1995 | 33 | PA | Yes | Yes | NA | 28.5 | 41.0 | NA | Small | Cysts |
| 5 | Levran D ^(7)^  2003 | NA | NA | No | Yes | NA | NA | NA | NA | Normal | Normal |
| 6 | Bianchi PH ^(8)^  2016 | 29 | PA | Yes | Yes | 8.2 | 13.7 | 16.5 | NA | NA | NA |
| 7 | Falhammar H ^(9)^  2018 | 42 | 12 | Yes | Yes | 21 | 17 | Elevated | NA | NA | Streak gonads |
| 8 | Kitajima M ^(10)^  2018 | 24 | PA | Yes | Yes | 7.9 | 6.5 | 13.5 | NA | Small | Cysts |
| 9 | Blumenfeld Z ^(11)^ 2021 | 24 | PA | No | Yes | 13.5 | 12.7 | 28.0 | NA | NA | NA |
| 10 | Xu Y ^(12)^  2022 | 24 | PA | No | Yes | 26.8 | 15.9 | 100.2 | 1.43 | NA | NA |
| 11 |  | 29 | 16 | No | No | 7.5 | 4.5 | 64.9 | NA | Small | Cysts |
| 12 | Jiang S ^(13)^  2022 | 31 | 15 | No | No | 6.5 | 4.5 | 6.68 | 6.70 | Small | Cysts |
| 13 | Yang XL ^(14)^  2022 | 28 | 14 | No | NA | 5.8 | 7.8 | 16.25 | NA | NA | Cysts |
| 14 | Xi S ^(15)^  2023 | 35 | 14 | No | NA | 6.6 | 7.4 | 28.93 | 2.85 | Normal | NA |
| 15 | Pan P ^(16)^  2023 | 29 | 14 | No | NA | 7.5 | 3.3 | 41.9 | 3.19 | NA | Cysts |
| 16 |  | 31 | 14 | No | NA | 5.8 | 2.8 | 16.6 | 2.52 | NA | Normal |
| 17 |  | 29 | PA | No | NA | 3.3 | 3.8 | 0.91 ^c^ | 2.69 | NA | Normal |
| 18 |  | 28 | 14 | No | NA | 8.2 | 13.6 | 50.5 | 13.35 | NA | Cysts |
| 19 | Du X ^(17)^  2024 | 27 | 16 | No | Yes | 6.5 | 9.6 | 19.0 | NA | Small | Cysts |
| 20 |  | 32 | Yes ^d^ | No | NA | 4.6 | 4.0 | 16.7 | NA | Normal | Cysts |
| Pooled analysis ^e^ | | 29  (24 − 42) | 31.6% with PA | 25% with hypertension | 76.9% with adrenal insufficiency | 7.05  (3.3 − 26.8) | 7.8  (2.8 − 28.5) | 20.8  (6.7 − 100.2) | 2.77  (1.43 − 13.35) | 54.5% with small uterus | 75% with ovarian cysts |

a, Age at fertility treatment initiation; b, Adrenal insufficiency identified by baseline cortisol or peak plasma cortisol level < 18 μg/dL during adrenocorticotropic hormone stimulating test (4); c, With dexamethasone treatment; d, Reported without detailed number; e, Analyzed in cases reporting the corresponding data, presenting the median (minimum ~ maximum) values and the percentage of a certain symptom. AMH, anti-Mullerian hormone, FSH, follicle stimulating hormone; LH, luteinizing hormone; NA, not available; PA, primary amenorrhea.

# 5. Table S2. Assisted reproduction, reproductive and offspring outcomes in 20 patients with 17α-hydroxylase/17,20-lyase.

| **Case** | **Author and Publication year** | **Ovarian stimulation ^a^** | **Methods of conception** | **Number of embryo/**  **blastocyst transferred** | **Glucocorticoid**  **before transplantation** | **Progesterone suppression with GnRHa before transplantation** | **Progesterone before transplantation nmol/L** | **Pregnancy complications** | **Stress dose of glucocorticoids during delivery** | **Delivery outcome**  **and offspring follow-up** |
| --- | --- | --- | --- | --- | --- | --- | --- | --- | --- | --- |
| 1 | Our center | GnRHa ultra-long protocol | FET | 2 | DEX 0.375mg/d | Yes | 2.47 | HDP, GDM | Yes | CS at 37 wks GA;  Singleton live birth;  Healthy at five years old |
| 2 | Our center | GnRH antagonist regimen | FET | 2 | DEX 0.75mg/d | No | 1.32 | HDP, GDM | Yes | CS at 34 wks GA; Singleton live birth;  Neonatal Pneumonia;  Healthy at four years old |
| 3 | Our center | GnRHa short protocol | FET | 2 | PRED 5mg/d | Yes | 1.60 | HDP, Hypokalemia | NA | Embryonic demise at 16 wks |
| 4 | Ben-Nun I ^(6)^  1995 | Oocyte donation | FET | 4 | NA | No | NA | HDP, HELLP syndrome | Yes | Twin pregnancy,  CS at 26 wks GA; One died postnatally, the other was healthy at discharge after a  7-week NICU stay |
| 5 | Levran D ^(7)^  2003 | GnRHa long protocol | FET | ＞1 | DEX | Yes | NA | NA | NA | Triplet live births |
| 6 | Bianchi PH ^(8)^  2016 | GnRHa  long protocol | FET | 2 | DEX 0.5mg/d | Yes | 1.91 | HDP, GDM, ICP | NA | CS at 30 wks GA due to fetal distress; Singleton live birth |
| 7 | Falhammar H ^(9)^ 2018 | NA | NA | 3 | PRED 7.5mg/d | NA | NA | Shoulder Presentation | Yes | CS at 37 wks GA; Singleton live birth |
| 8 | Kitajima M ^(10)^  2018 | GnRHa long protocol | FET | 1 blastocyst each | DEX 0.5mg/d | No | <0.64 | NA | NA | Two consecutive live births; One with massive intrapartum hemorrhage |
| 9 | Blumenfeld Z ^(11)^ 2021 | GnRHa ultra-long protocol | FET | 2 | PRED 10mg/d | Yes | <1.20 | No | Yes | Induction of labor at 41 wks GA; Singleton live birth |
| 10 | Xu Y ^(12)^  2022 | PPOS | FET | NA | PRED 5mg/d | Yes | NA | No | NA | Term singleton live birth |
| 11 |  | PPOS | FET | 2 | DEX 0.75mg/d | No | NA | No | NA | Term singleton live birth |
| 12 | Jiang S ^(13)^  2022 | PPOS | FET | 2 | DEX 0.75mg/d | No | NA | No | NA | Term singleton live birth |
| 13 | Yang XL ^(14)^  2022 | PPOS | FET | 2 | DEX 0.375mg/d | Yes | 0.67 | GDM | NA | Twin live birth at 35 wks GA; healthy at one years old |
| 14 | Xi S ^(15)^  2023 | PPOS | FET | 2 | DEX 0.375mg/d | No | 1.81 | No | NA | CS at 39 wks GA due to fetus breech position; Singleton live birth; Healthy at one year old |
| 15 | Pan P ^(16)^  2023 | GnRHa long protocol | FET | 2 | No | Yes | NA | NA | NA | Pre-term singleton live birth |
| 16 |  | GnRHa long protocol | FET | 3 | DEX 0.75mg/d | Yes | 2.13 | NA | NA | Pre-term singleton live birth; CS |
| 17 |  | PPOS | FET | 2 | DEX 0.75mg/d | Yes | ＜0.10 | NA | NA | Term singleton live birth; CS |
| 18 |  | GnRHa ultra-long protocol | FET | 2 | DEX 0.75mg/d | No | 2.23 | NA | NA | Term singleton live birth; CS |
| 19 | Du X ^(17)^  2024 | Timed intercourse，natural pregnancy | NA | NA | PRED 7.5mg/d | NA | 3.34 | NA | NA | Term singleton live birth |
| 20 |  | GnRHa down-regulation protocol | FET | 1 | PRED 7.5mg/d | No | 3.34 | No | NA | Term singleton live birth; CS |
| Pooled analysis ^b^ | | Details in the legend ^c^ | 1 natural conception, 22 IVF-FET | 1 − 4 | 94.7%  (13 with DEX,  5 with PRED) | 52.6%  (10/19) | 1.81  (< 0.64 − 3.34) | 46.2%  (6/13) | 5 cases | Five term vaginal deliveries (25%) |

a, The ovarian stimulation protocol for cycles achieving clinical pregnancy; b, Analyzed in cases reporting the corresponding data, presenting the median (minimum ~ maximum) values and the percentage of a certain symptom; c, 9 GnRHa down-regulation protocol, 1 GnRH antagonist regimen, 1 GnRHa short protocol, 6 PPOS, 1 oocyte donation, 1 natural pregnancy, and 1 unreported. CS, cesarean section; DEX, dexamethasone; FET, frozen embryo transfer; GA, gestational age; GDM, gestational diabetes mellitus; GnRHa, gonadotropin-releasing hormone agonist; HDP, hypertensive disorders of pregnancy; ICP, Intrahepatic Cholestasis of Pregnancy; IVF-FET, in vitro fertilization - frozen embryo transfer; NA, not available; PPOS, Progestin-primed ovarian stimulation; PRED, Prednisone.

# 6. Clinical manifestations and diagnostic processes of the three patients from our center

**Case 1:** She was a 34-year-old female. Her menarche occurred at age 18, with a menstrual cycle of 28–35 days. Four years ago, she was diagnosed with an ovarian cyst at another hospital due to infertility and underwent laparoscopic ovarian cystectomy. She had no special family history. Physical examination: blood pressure 113/69 mmHg, height 170 cm, weight 71 kg. Breast development was in Tanner stage V and pubic hair development was in Tanner stage II with no axillary hair. Laboratory tests in follicular phase showed elevated progesterone (10.9 nmol/L). Her FSH, LH, estradiol, 17OHP, cortisol, ACTH, and blood potassium levels were within the normal ranges. The peak cortisol level in the ACTH stimulation test was 14.2 μg/dL. Ultrasound showed a normal uterus, but two cysts were observed in the right ovary (the larger one 2.6 × 1.2 cm), and 8–9 small follicles were seen in the left ovary. Genetic testing revealed compound heterozygous mutations in the *CYP17A1* gene (Table S1), confirming a diagnosis of 17OHD.

**Case 2:** A 24-year-old female presented with oligomenorrhea since menarche at age 12. At age 19, she was diagnosed with hypertension (190/140 mmHg) and hypokalemia (3.3 mmol/L). Genetic testing confirmed 17OHD due to compound heterozygous *CYP17A1* mutations (Table S1). Initial treatment with dexamethasone 0.75 mg before bed and nifedipine 30 mg daily normalized her blood pressure and electrolytes. During fertility preparation three years ago, other hospital prescribed increased dexamethasone doses (0.75–3 mg/day) but failed to suppress her markedly elevated progesterone (7.4–46.2 nmol/L), and led to Cushingoid features. Her two younger sisters also had 17OHD (with 46, XX and 46, XY karyotypes respectively) and shared the same *CYP17A1* mutations as the proband. Her parents were healthy. Physical examination showed her BP 130/85 mmHg, height 163 cm, weight 70 kg with apparent abdominal striae. Her breasts were in Tanner IV, and pubic hair was in Tanner I. Laboratory tests showed her follicular phase progesterone was 31 nmol/L. Her FSH, LH, estradiol, and potassium were normal. Ultrasound showed small uterus, bilateral ovaries with 3–4 follicles (0.2–0.9 cm), and left ovarian cysts (largest 4.0 × 2.8 cm). The adrenal MRI was normal.

**Case 3**: She was a 30-year-old female. She experienced menarche at age 14 with regular 30-day cycles. Her menstrual cycles became irregular (10 to 90 days) at age 24 after a period of psychological stress. She had a 3-year history of infertility and underwent three unsuccessful cycles of ovulation induction with timed intercourse at another hospital. There is no similar family history. In physical examination, her had BP 120/86 mmHg, height 160 cm and weight 66 kg. Breast development was in Tanner stage V, and pubic hair was in Tanner stage III. Laboratory findings found her follicular-phase progesterone (20.8 nmol/L) greatly increased with a slightly elevated FSH (8.1 mIU/mL) and (LH 14.3 mIU/mL). Her estradiol, cortisol, ACTH, and serum potassium were within normal ranges. An ACTH stimulation test showed a peak cortisol of 15.0 ng/ml, suggesting an impaired adrenal function. Ultrasound found the uterus smaller than normal. Both ovaries showed 6–9 follicles (0.2–0.9 cm), along with multiple cysts (largest 4.2 × 3.0 cm). Whole-exome next-generation sequencing and third-generation sequencing both identified a heterozygous, pathogenic mutation in the *CYP17A1* gene: c.1394T>C (Table S1). Sanger sequencing confirmed the mutation was inherited from the father, with a wild-type genotype from the mother who had no symptoms. Although we haven’t identified a second mutation, based on the characteristic clinical manifestations, we still consider the diagnose of 17OHD.

# 7. Table S3. *CYP17A1* Genotypes of the three patients from our center.

| **Case** | **Gene sequencing method** | **Mutation** | **From** | **Pathogenicity assessment** |
| --- | --- | --- | --- | --- |
| 1 | Second generation whole-exome sequencing | c.1263G>A | Father | Pathogenic |
|  |  | c.1459_1467delGACTCTTTC | Mother | Pathogenic |
| 2 | Second generation whole-exome sequencing | c.1169C>G | Mother | Pathogenic |
|  |  | c.548G>A | Father | Likely pathogenic |
| 3 | Third generation whole-exome sequencing | c.1394T>C | Families unwilling to get tested | Pathogenic |
